# Transcriptional analyses reveal the molecular mechanism governing shade tolerance in the invasive plant *Solidago canadensis*

**DOI:** 10.1002/ece3.6206

**Published:** 2020-03-24

**Authors:** Miao Wu, Zeyu Li, Jianbo Wang

**Affiliations:** ^1^ College of Life Sciences Wuhan University Wuhan China

**Keywords:** gene expression, invasion, photosynthesis, shade stress, *Solidago canadensis*

## Abstract

*Solidago canadensis* is an invasive plant that is capable of adapting to variable light conditions. To elucidate the shade tolerance mechanism in *S. canadensis* at the molecular level, transcriptome analyses were performed for leaves growing under natural light and three shade level conditions. Many differentially expressed genes (DEGs) were found in the comparative analysis, including those involved in photosynthesis, antioxidant, and secondary metabolism of phenol‐ and flavonoid‐related pathways. Most genes encoding proteins involved in photosynthesis, such as photosystem I reaction center subunit (Psa), photosystem II core complex protein (Psb), and light‐harvesting chlorophyll protein (Lhca and Lhcb), and reactive oxygen species (ROS) scavenging‐related enzymes, such as superoxide dismutase (SOD), peroxidase (POD), and catalase (CAT), were upregulated with the shade levels. Furthermore, most of the DEGs related to secondary metabolite synthesis were also upregulated in the shade conditions. Our study indicates that *S*. *canadensis* can respond to shade stress by modulating the expression of several photosynthesis‐related, free radical scavenging‐related, and secondary metabolism‐related genes; thus, this species has the ability to adapt to different light conditions.

## Graphical abstract

The invasive plants are widely distributed in different external environments and have a good adaptive ability. Through the analysis of gene expression in the leaves of *Solidago canadensis* under different shading conditions, the genes that played an important role in responding to shading stress were screened out. The differential expression of these genes may play an important role in the response of *S. canadensis* plant to light avoidance environment. It is possible to cope with different light intensities, which are likely to play an important role in the process of *S. canadensis* expanding invasion and forming invasive species. At the same time, it also provides a theoretical basis for the distribution of *S. canadensis*.

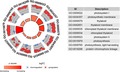

## 1 INTRODUCTION

Biological invasion represents a threat to ecosystem management. Many studies have revealed that invasive plants have the ability to adapt to the scarcity in resources such as availability of nutrients, water, light, and various environmental stresses. (te Beest et al., 2012; Colautti & Barrett, 2013; Rosche et al., 2017; Thébault, Müller‐Schärer, & Buttler, 2011). Understanding how these plants can invade an ecosystem has therefore been a major challenge for ecologists (Simberloff et al., 2013). Recently, biological invasion has received increased attention in terms of the factor(s) promoting invasion success (Bock et al., 2015; Pandit, White, & Pocock, 2014). Consequently, a growing emphasis has been placed on elucidating the mechanisms that lead to higher capabilities of invasiveness in invasive species (Pandit et al., 2014). Invasive plants frequently possess broader ecological amplitudes and greater plasticity (te Beest et al., 2012; Si et al., 2014), while increasing evidence suggests a higher phenotypic plasticity, which acts as an important determinant for species invasiveness (Amina et al., 2014; Molina‐Montenegro et al., 2013). On the one hand, phenotypic plasticity allows the plant to respond to altered environmental conditions by changing its phenotype, a phenomenon that has been recorded for many species that assume different response traits in variable environments (Ortega‐Mayagoitia, Hernandez‐Martinez, & Ciros‐Perez, 2018). This characteristic plays a central role in biological invasions by permitting individuals to colonize different environments and establish viable populations (Du, Liu, Yan, Li, & Li, 2017).

Light is one of the most prominent environmental factors for plant growth (Wang, Chen, Botella, & Guo, 2019). Light not only affects the rate of photosynthesis, antioxidant enzyme activity, and the production of biomass, but also it also regulates all aspects of development in plants (Li, Xin, et al., 2017; Wu, Chen, Sun, Deng, & Chen, 2016). Shade can cause certain metabolic changes as well as imbalances in photosynthesis and carbohydrate production, potentially limiting plant growth and stability (Huang, Zhang, & Liu, 2018; Sajad et al., 2018). Plants are able to respond to changes in light decreased conditions by adjusting a range of physiological and morphological characteristics, such as the specific leaf area, leaf size, biomass allocation pattern, and chlorophyll content (Feng & van Kleunen, 2014; Legner, Fleck, & Leuschner, 2014; Liu et al., 2016). Plants can respond to light decreased environments by altering gene expression levels, for example, photosystem‐related genes are differentially expressed, and most transcription factor (TF) genes are induced under shade conditions in *Camellia sinensis* (Wu et al., 2016). Gibberellin biosynthesis genes are found to play a role in shade tolerance in *Lolium perenne* (Li, Katin‐Grazzini, et al., 2017). The expression of genes related to photosystem I and photosystem II could be affected by various light conditions (Rogowski et al., 2019). Shade avoidance and tolerance responses in plants could be reflected in the alteration of expressed genes, such as those involved in hormone signaling and pigment biosynthesis (Ranade, Delhomme, & García‐Gil, 2019).

Invasive plants can spread across diverse habitats via individual plasticity (Williams, Mack, & Black, 1995). When dispersed in a shaded environment with low light intensities, invasive plants frequently develop a greater leaf area and biomass to enhance their light capture and utilization efficiency to adapt to the reduced light conditions (Du et al., 2017). This phenomenon reveals that plants have developed some regulatory processes to adapt to shade conditions and may enable their invasion into wider areas.

*Solidago canadensis* L. (Asteraceae), native to North America, has successfully invaded many areas worldwide. The success of invasion can be related to the plant's high phenotypic plasticity (Du et al., 2017). *S*. *canadensis* has a relatively wide niche with different soil properties in Europe (Szymura & Szymura, 2013) and can rapidly spread in non‐native regions with different climatic conditions (Li, Du, Guan, Yu, & van Kleunen, 2016). Currently, *S*. *canadensis* has been naturalized in many areas and is considered one of the most destructive and widespread invasive species (Wang, Jiang, Zhou, & Wu, 2018), distributed in multiple areas, including farmland, the sides of roads, and upland forests.

To date, the biological processes of *S*. *canadensis* in response to shading stress have been unclear. The present study provides a comprehensive description of the differential gene expression patterns in this plant under different degrees of shading. We examine the consequences of three different light intensity levels of shade treatment on the morphological, physiological, and transcriptional characteristics of *S*. *canadensis*. Based on the comprehensive analysis, candidate shade stress‐responsive genes are identified in comparison with control natural light conditions. These findings will contribute to understanding how *S*. *canadensis* adapts to the shade environment and may help to elucidate how it has become an invasive species.

## 2 MATERIALS AND METHODS

### 2.1 Plant materials and treatment

**Figure 1:**
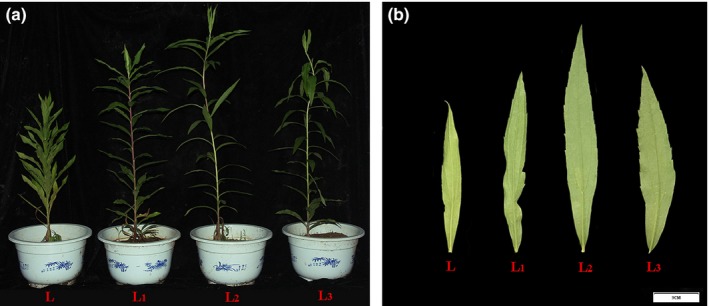
The appearance of *S. canadensis* cultivated under various levels of shade treatments. (a) The phenotype of each whole plant; (b) the phenotype of each leaves

In this study, we transplanted 36 rhizomes, respectively, originated from 36 plants of the hexaploid *S*. *canadensis* growing at the suburb of Wuhan city to the open air garden in Wuhan University (30°32′N, 114°25′E), Hubei Province, China. After three months (from February to April) of cultivation in pots under natural conditions (Figure 1), we selected 12 of the 36 plants with similar growth status and randomly divided them into four groups. One of these groups was used as control, and the other three groups were covered with one layer, two layers, and three layers of shade net, respectively. In the control treatment (L), plants were cultivated under natural light. In the three different levels of shade treatment with one‐, two‐ and three‐layer shade nets, the natural irradiance availability was reduced to 50% (L_1_), 25% (L_2_), and 10% (L_3_), respectively, via black shade nets that were clamped to a metal frame at a height of 150 cm (Semchenko, Lepik, Gotzenberger, & Zobel, 2012). Light intensities were measured by a handheld luxmeter (SMART Digital Lux Meter AS 813). After cultivation for 21 days, we collected leaves from the four treatment groups, with each containing three replicates. The leaves in each plant were collected from the top third to fifth leaves, and these leaves were prepared for RNA extraction, quantitative real‐time PCR analysis, and chlorophyll content measurement.

### 2.2 Measurement of chlorophyll content

One hundred milligrams of each fresh leaf sample from the four treatment was ground with a sterile pestle and morter. The samples were transferred into 15‐ml tubes followed by the addition of 10 ml 96% ethanol. After mixing well, the tubes were placed at 4°C for two days. The samples were centrifuged at 17925 *g* at 4°C for 1 min, and then, the OD value of the supernatant was determined at wavelengths of 470, 665, and 649 nm with an MAPADA UV‐1200 spectrophotometer (Shanghai Meipuda Instrument Co. Ltd.). The contents of chlorophyll *a*, *b*, and total carotenoid for all samples were calculated according to previous studies as follows: *C*_a_ = 13.95 × A665–6.88 × A645; *C*_b_ = 24.96 × A649–7.32 × A665; *C_x_*_+c_ = (1,000A470–2.05*C*_a_–114.8*C*_b_)/245 (Lichtenthaler & Wellburn, 1985).

### 2.3 mRNA isolation, library construction, and RNA‐seq

The leaves of 12 samples from the four treatments were collected for mRNA isolation. After being fully ground, total RNA from frozen leaves was isolated using TRIzol reagent. RNA integrity and purity were checked using an Agilent 2100 Bioanalyzer (Agilent RNA 6000 Nano Kit) with a minimum integrity value of 7.5. The cDNA library of each sample was constructed as follows. First, mRNA was isolated from total RNA with magnetic beads containing Oligo (dT) primer. Subsequently, the enriched mRNAs were fragmented in fragmentation buffer, and then, first‐strand and second‐strand cDNA were synthesized and amplified using an ABI Step One Plus Real‐Time PCR System. Finally, these cDNA library preparations were sequenced using an Illumina HiSeq X Ten from both the 5′ and 3′ ends, and 150‐bp paired‐end reads were generated.

### 2.4 De novo assembly and functional annotation

Raw reads containing low‐quality, adaptor‐polluted, and a high content of unknown bases were removed and satisfied with the number of Q20 above 90% before downstream analyses. After filtering out low‐quality and ambiguous nucleotides, de novo assemblies were prepared from the clean reads using Trinity (v2.0.6; Grabherr et al., 2011). A transcriptome assembly, generated from all mixed samples, was used as a reference for further gene expression analysis. For gene function annotations, all nonredundant assembled transcripts (≥200 bp) were used to search against public databases, including the nonredundant protein (Nr), nucleotide (Nt), EuKaryotic Orthologous Groups (KOG), Swiss‐Prot, and Kyoto Encyclopedia of Genes and Genomes (KEGG) databases, with a significance threshold of *E*‐value ≤ 10^−5^. Based on the NR annotation results, Blast2GO software was used to classify unigenes against the Gene Ontology (GO) database according to cellular component, biological process, and molecular function (Gotz et al., 2008).

### 2.5 Differentially expressed gene analysis

All clean reads from each sample were mapped to the assembled unigene dataset with Bowtie2 software (Langmead & Salzberg, 2012), and the gene expression levels were calculated with RSEM software (Li & Dewey, 2011). To provide a relatively accurate assessment of transcript abundance, the fragments per kilobase of exon model per million mapped reads (FPKM) value were used as a measure to normalize the unigene expression levels (Mortazavi, Williams, Mccue, Schaeffer, & Wold, 2008). The differentially expressed genes (DEGs) between each group were identified with DEseq2 software with a |log_2_ (fold change)| ≥ 1 and adjusted *p*‐value < .05 as the threshold for significantly different expression (Love, Huber, & Anders, 2014). GO enrichment analysis of all DEGs was performed using hyper, a function of R based on the hypergeometric distribution (Young, Wakefield, Smyth, & Oshlack, 2010). Furthermore, REVIGO was used to visualize the enriched GO term in multiple ways to facilitate interpretation (Supek, Bosnjak, Skunca, & Smuc, 2011). For KEGG pathway enrichment analysis, all DEGs were mapped to pathways in the KEGG database and identified significantly enriched pathways based on a corrected *p*‐value < .05.

### 2.6 Gene expression network construction

Weighted gene correlation network analysis (WGCNA) is used to construct the expressed gene network and identify stress‐related candidate key genes. This method has been widely applied in model organisms and nonmodel organisms for gene expression studies (Fu et al., 2014; Lou et al., 2017). The gene network was constructed using the R package WGCNA (Langfelder & Horvath, 2008), and all the DEGs between the shade treatment and control were applied to WGCNA. These collected genes were clustered to form different modules, which were assembled by unique color. A significant correlation for each module was satisfied with coefficients >0.80 and *p*‐values < .01. Modules that were significantly correlated with shade stress were extracted and exported into Cytoscape (ver. 2.8, http://www.cytoscape.org/) for network visualization (Smoot, Ono, Ruscheinski, Wang, & Ideker, 2011). Hub genes were identified based on the connection degree of each node in the network.

### 2.7 Statistical analysis

In order to investigate whether the chlorophyll and carotenoid content of plants were changed under different light conditions, the experiment of chlorophyll content measurement for each treatment was repeated three times in this study. The data of chlorophyll and carotenoid content were analyzed by one‐way analysis of variance, and the mean (±*SD*) was the average calculated from three biological replicates. Letters on the bars represent a significant difference at *p*‐value ≤ .05 (LSD test) among different treatments compared with the control group.

### 2.8 Validation of gene expression by qRT‐PCR

The quantitative real‐time polymerase chain reaction (qRT‐PCR) technique was used to validate gene expression from RNA‐Seq. RNA was isolated with TRIzol reagent from each independent biological sample. In order to prevent the presence of DNA in extracted RNA samples, all the RNA samples were treated with DNase I to digest DNA. cDNAs were obtained by reverse transcription of each RNA sample. Specific primers for 12 randomly selected genes were designed using Primer 5 software (Lalitha, 2000). The SYBR Green Master Mix (Applied Biosystems) was used by quantitative PCR. The relative expression of selected genes was normalized by the 2^−∆∆^ *^Ct^* method based on the 18S ribosomal gene as a reference.

## 3 RESULTS

### 3.1 Changes in morphological and chlorophyll content under shade stress

**Figure 2:**
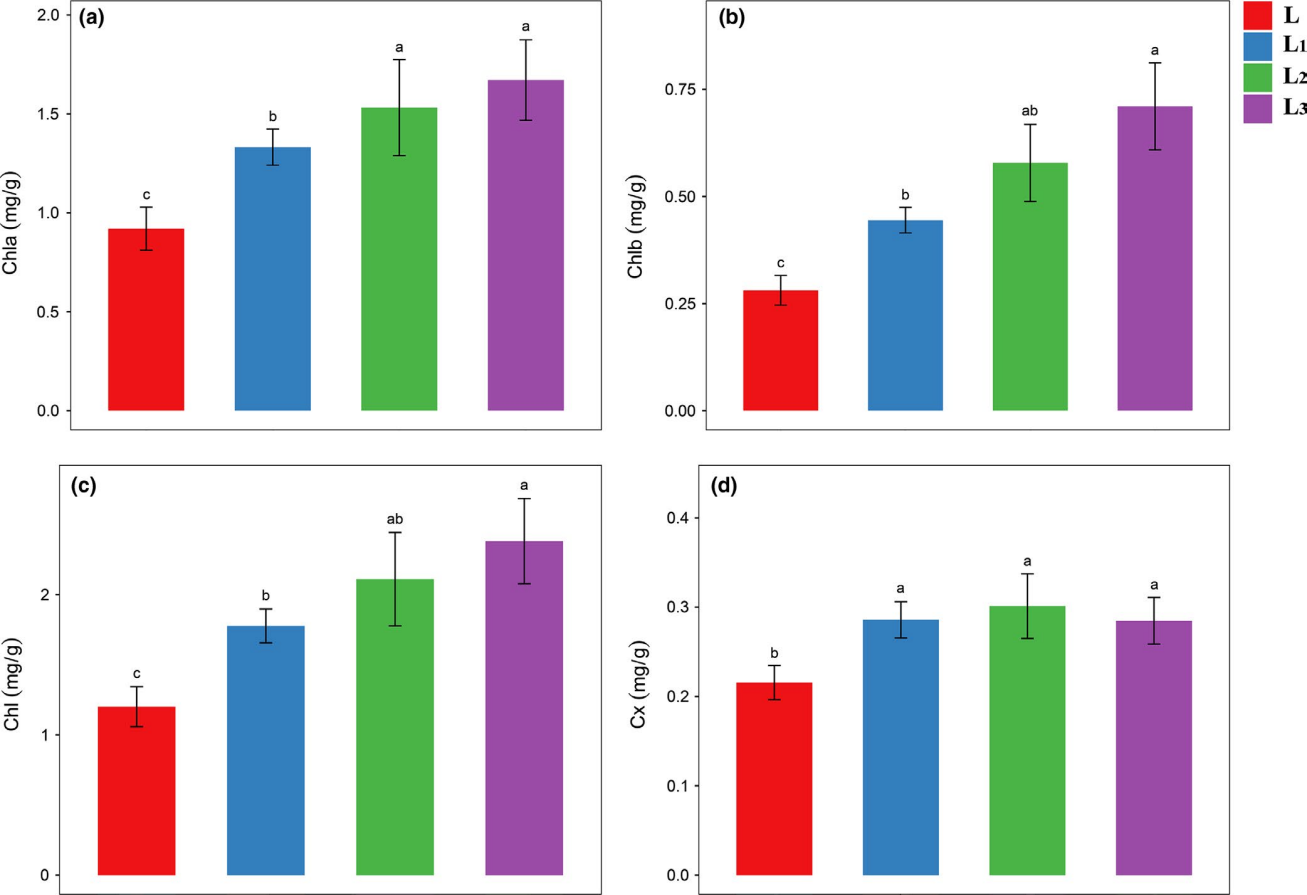
The content of pigment in various levels of shade treated leaves. (a) Chl *a*; (b) Chl *b*; (c) total of Chl; and (d) carotenoid. The values represented mean ± *SE*, and different letters mark significant differences among shade treatment groups (*p* < .05). The red color bar represents L group (control treatment), and the blue, green, and purple color bar represent L_1_ (one‐layer shade net condition), L_2_ (two‐layer shade net condition), and L_3_ (three‐layer shade net condition) group, respectively

To investigate how shade affected *S. canadensis* growth, changes in plant morphology was observed under three treatment groups in comparison with control plants. Three levels of shading treatment significantly increased the plant height, leaf angle (Figure 1a), and leaf length (Figure 1b) compared with the control. Interestingly, the two‐layer shading net (L_2_)‐treated plants had the highest growth height and largest leaves. The plant height and leaf area of the three‐layer shading net (L_3_) were smaller than those of the L_2_ but higher than those of the one‐layer shading net (L_1_) treatment groups. In the three different shading treatment groups, the leaf angle and total content of chlorophyll increased with the reduction of light intensity and were significantly higher than the control group. In addition, there was a significant difference in total chlorophyll content between the L with L_1_, L_2_, and L_3_ groups (Figure 2c). The content of chlorophyll a and chlorophyll b also increased with the reduction of light intensity, and most groups were also significantly different from each other (Figure 2a,b). The carotenoid content in the three treatment groups increased compared with the L group, while there were no significant differences among these three groups (Figure 2d). In contrast, the ratio of Chl *a*/Chl *b* and carotenoid/total Chl in each group decreased with reduced light intensity (Table S1). These results suggest that *S. canadensis* can exhibit different phenotypes and pigment levels under shade stress compared with natural growth condition.

### 3.2 Overview of transcriptome profiles

To understand the molecular basis of the phenotypic differences in shade conditions, we generated transcriptome profiles based on RNA‐Seq analyses. The leaf samples of three different shade conditions and one natural light condition were used for RNA‐Seq analysis with three biological replicates. In total, 12 libraries were constructed and sequenced on an Illumina HiSeq X Ten platform, with each library satisfied with a Q20 percentage greater than 98% (ranging from 98.41% to 98.82%) and single read length of 150 bp (Table S2). The clean reads were assembled into 123,598 unigenes with an average length of 1,029 bp and N50 length of 1,443 bp. The sequencing data have been deposited in the Sequence Read Archive of the National Center for Biotechnology Information (accession number: PRJNA577291). The assembled unigene sequences were aligned by searching the Nr, Swiss‐Prot, COG, and GO public databases (Table 1). Based on the annotated unigenes, 76,060, 74,710, 74,406, and 77,965 unigenes were identified to be expressed by FPKMs in the L, L_1_, L_2_, and L_3_ group, respectively. By redundancy analysis of annotated genes from these four groups, a total of 83,575 unigenes were identified, with 67,237 unigenes detected in all four groups and 1,204 unigenes that were unique to the L group, 592 to the L_1_ group, 651 to the L_2_ group, and 2,488 to the L_3_ group (Figure S1). The expressed genes were divided into four ranges according to the FPKM, and approximately 21.3% of the genes were expressed in the range of 0–1, 41.3% in the range of 1–5, 30.7% in the range of 5–30, and 6.7% were above 30.

**Table 1:** Summary of annotations of the *S. canadensis* transcriptome

Annotation database	Number of annotated unigenes	Percentage of all‐unigenes (%)
Nr	83,174	67.29
Nt	44,666	36.14
Swiss‐Prot	62,104	50.25
KEGG	59,538	48.17
COG	28,873	23.36
GO	53,774	43.51
Total number of all‐unigenes	123,598	

### 3.3 Comparison of DEGs under shade conditions

**Figure 3:**
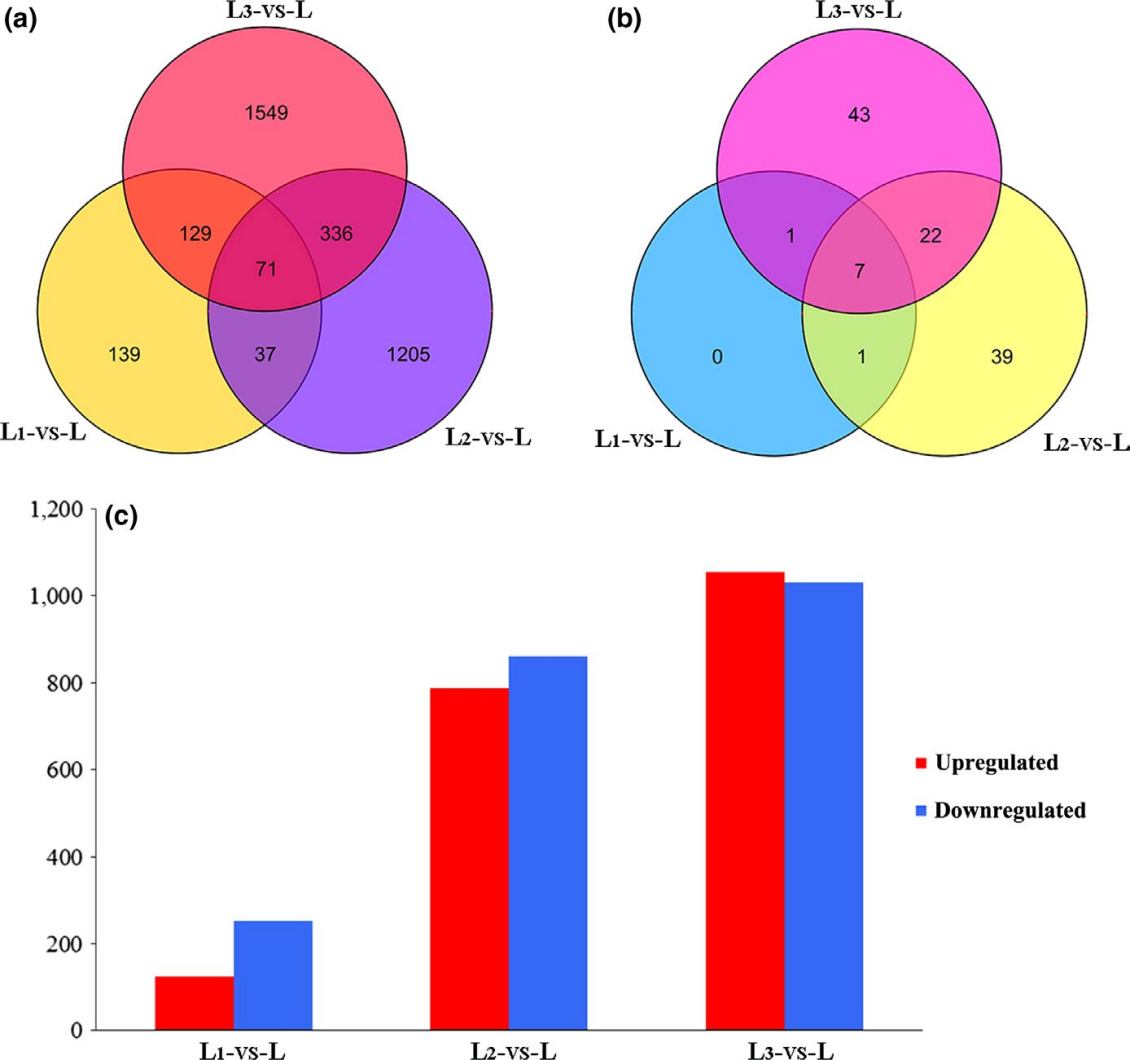
The number of DEGs in three comparison groups. (a) Venn diagram of all DEGs; (b) Venn diagram of differentially expressed TF genes; (c) the number of up‐ and downregulated DEGs

**Figure 4:**
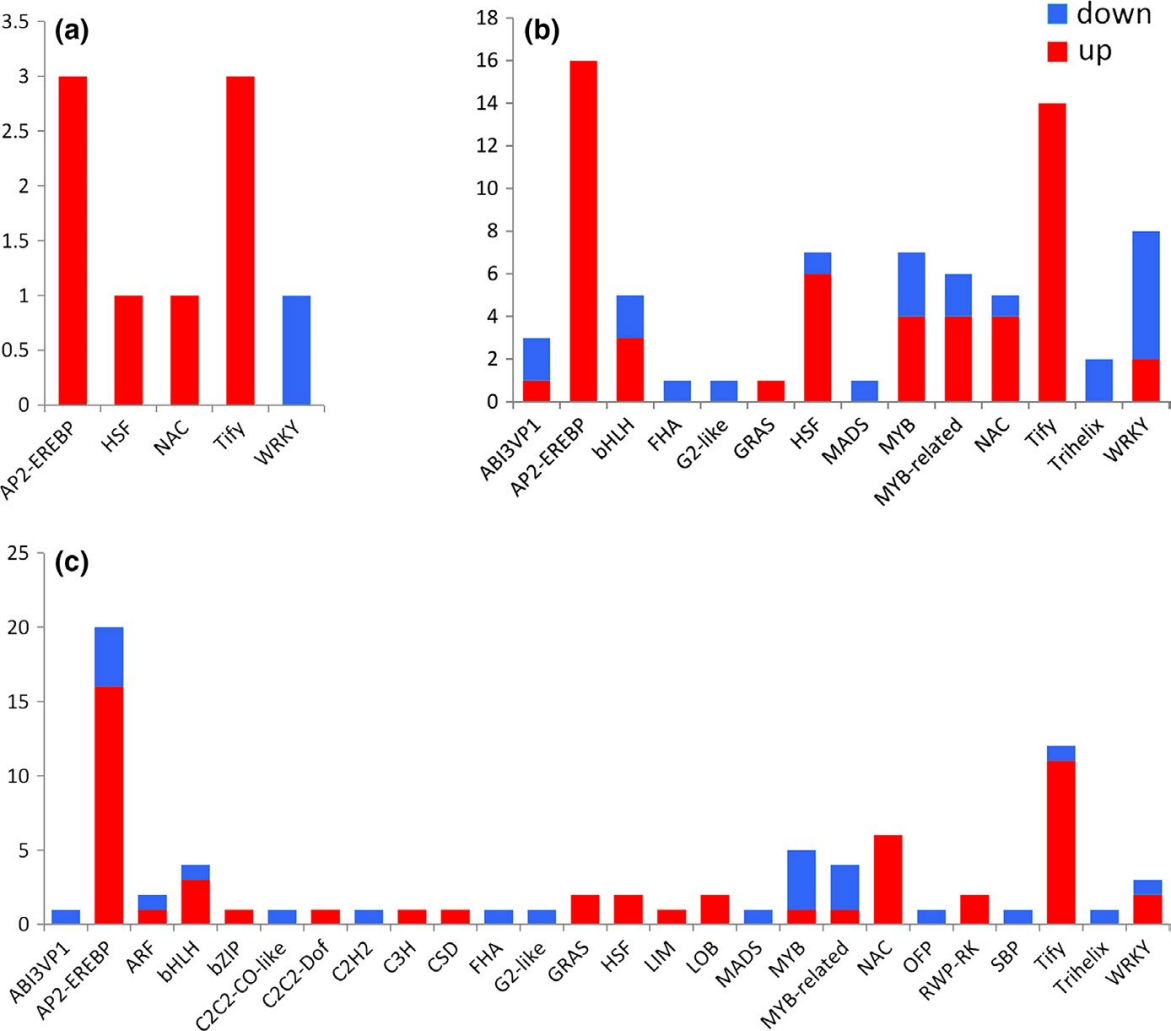
The number of up‐ and downregulated TF genes in three comparison groups. (a) L_1_ versus L group; (b) L_2_ versus L group; and (c) L_3_ versus L group

When we examined the number of DEGs for each of these three comparisons (Figure 3c), we observed that the reduced light intensity condition had a greater impact on gene expression (i.e., more DEGs) in *S*. *canadensis*. There were 376 DEGs in the comparison of L_1_ versus L, with 124 being upregulated and 152 downregulated. Similarly, there were 1,649 DEGs (788 upregulated and 861 downregulated) in the L_2_ condition comparison and 2,087 DEGs (1,054 upregulated and 1,031 downregulated) in the L_3_ condition compared with their light‐grown counterparts (Figure 3c). According to the overlap of DEGs among these three comparison groups, 71 DEGs appeared in all three groups; in addition, 139 unique DEGs were identified in the L_1_ versus L group, 1,205 in L_2_ versus L, and 1,549 in L_3_ versus L (Figure 3a). From each of comparison, nine DEGs were categorized into five TF gene families in L_1_ versus L (Figure 4a), and 69 DEGs were categorized into 14 TF gene families in L_2_ versus L (Figure 4b), and 73 DEGs were categorized into 26 TF gene families in L_3_ versus L (Figure 4c). The number of differentially expressed TF genes increased as the degree of shading stress increased. Nine transcription factor genes were differentially expressed in L_1_ versus L, and all of them were included in the other two comparison groups, while seven TF genes were also expressed in the other two comparison groups. Moreover, 29 differentially expressed TF genes were coexpressed in L_2_ versus L and L_3_ versus L, with 39 and 43 specifically expressed in L_2_ versus L and L_3_ versus L, respectively (Figure 3b). Most of these differentially expressed TF genes were upregulated; the *AP2‐EREBP* and *Tify* TF genes were most predominant in each comparison group followed by the *MYB*, *WRKY,* and *bHLH* TF genes (Figure 4). These results showed that the expression level of shading stress response genes in *S*. *canadensis* was increased with reduced light intensity. In addition, the regulation of these DEGs may participate in an acclimation strategy to different light conditions in *S*. *canadensis*.

### 3.4 GO functional enrichment analysis

**Figure 5:**
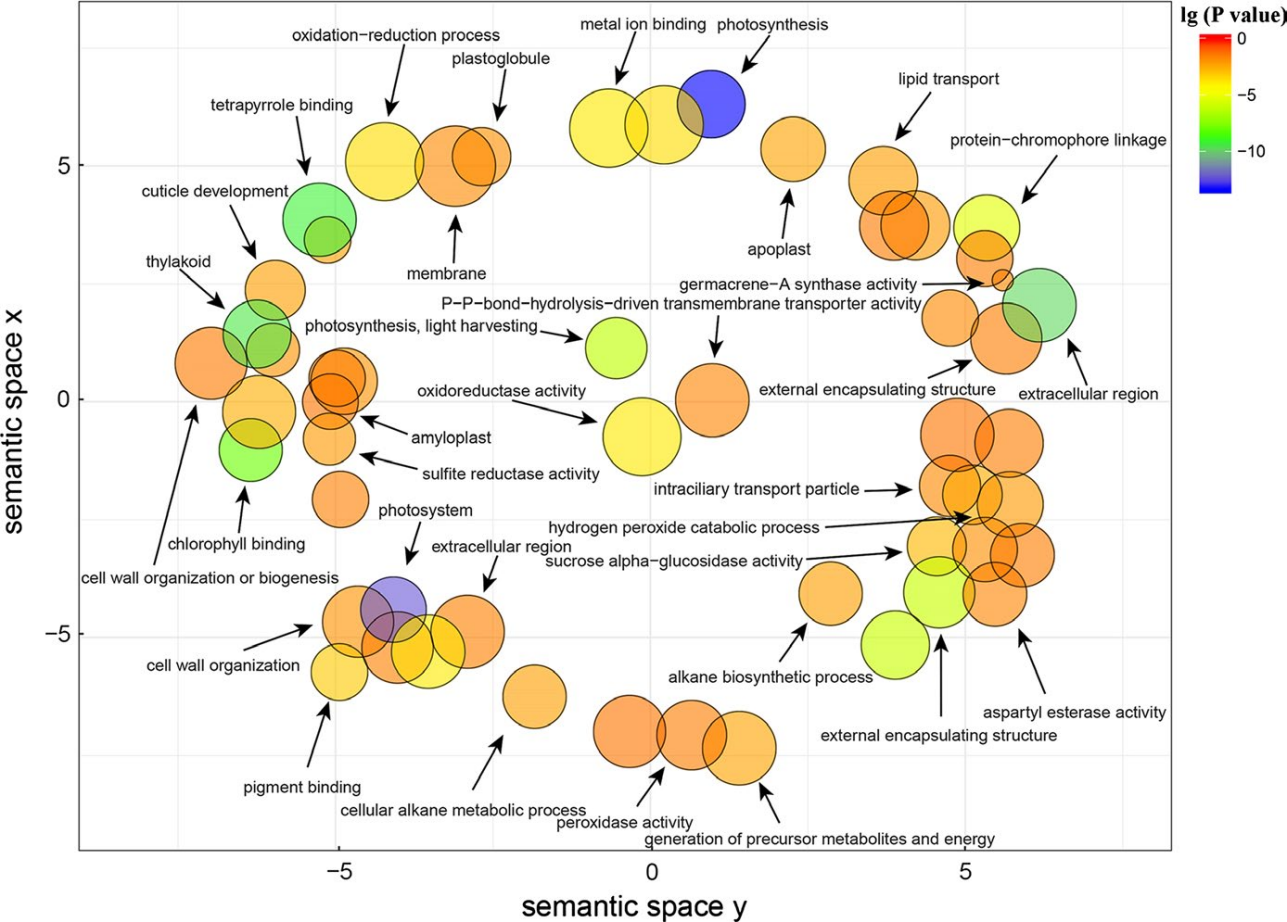
The result of Gene Ontology (GO) analysis by REVIGO. The log10 values of the *p*‐value for each cluster were represented based on the color gradation. The bubble size indicates the frequency of the GO term in the underlying GO database (bubbles of more general terms are larger)

**Figure 6:**
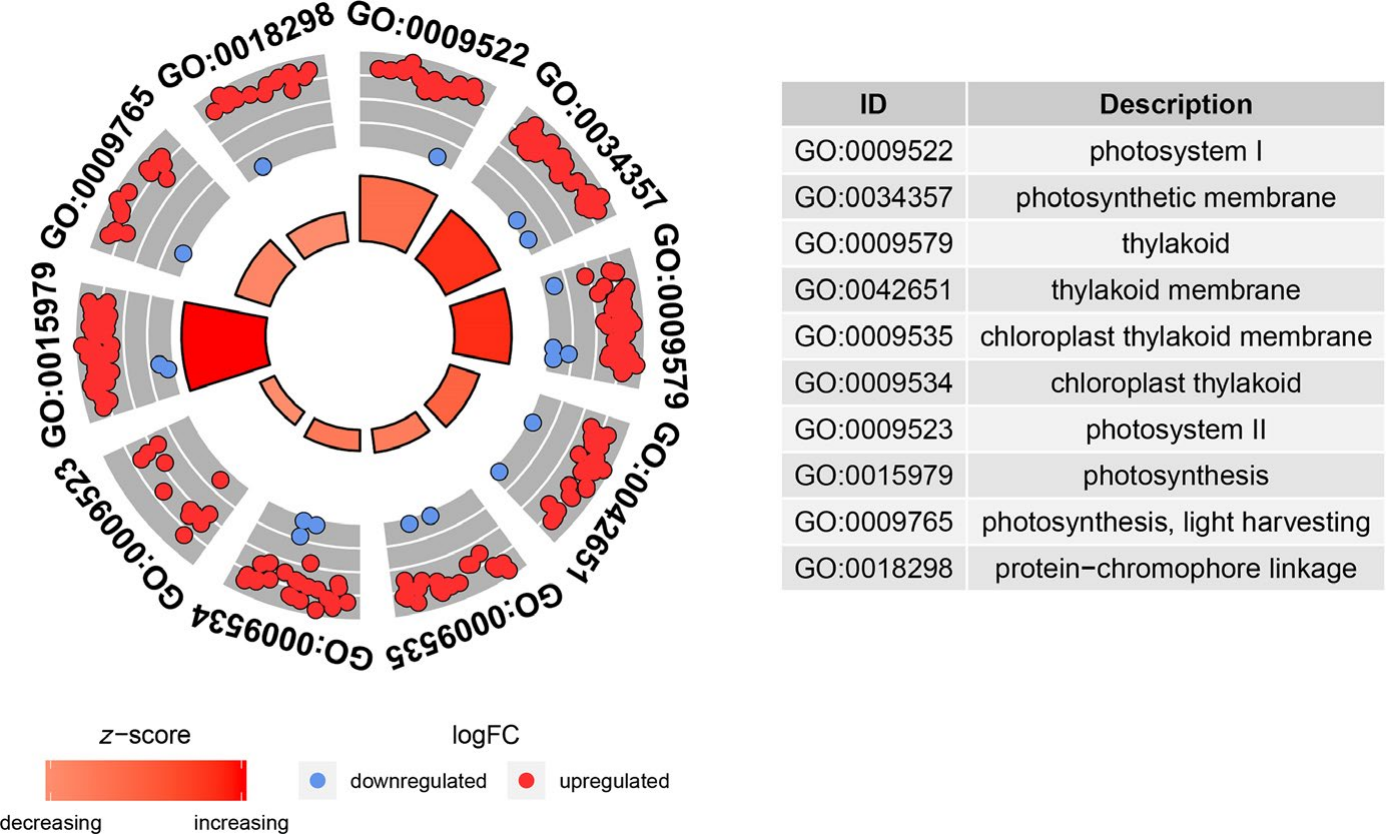
GO circle plot displaying gene annotation enrichment analysis. Radar chart shows the distribution of individual terms in the annotation categories. The fold changes (FC) of gene expression values (log2 FC) were derived from three biological replications corresponding to each sample. Within each selected GO term, blue dot shows a gene downregulated at shading stress and red dot indicates a gene upregulated at shading stress. The outer to inner layers of gray circles indicate the relative fold change of gene expression (from higher to lower). The height of the inner rectangle represents the *p*‐value of the GO term. The rectangles were colored with the red gradient according to the z‐score. (*p* < .05, FDR adjusted *p* < .05) $Z\;-\;\text{score}=\left(\text{upregulated}-\text{downregulated}\right)/\sqrt{\text{upregulated}+\text{downregulated}}$

To further explore the function of DEGs, GO functional enrichment was conducted. A total of 3,466 DEGs from the three compared groups were evaluated with GO enrichment and enriched into 49 GO terms classified into three main GO categories (Figure S2). Many genes were significantly enriched in GO terms related to photosynthesis, reactive oxygen species (ROS), and secondary metabolism synthesis (Table S3). For example, in the category of cellular component, GO terms, such as “photosystem,” “photosynthetic membrane,” “thylakoid part,” “photosystem I,” “photosystem II” and “chloroplast,” which related to the photosystem were significantly enriched. In the category of molecular function, GO terms, such as “germacrene‐A synthase activity,” “pigment binding,” “peroxidase activity” and “sesquiterpene synthase activity,” which related to ROS response and secondary metabolite synthesis were significantly enriched. In the category of biological processes, GO terms, such as “photosynthesis,” “photosynthesis, light harvesting,” “protein‐chromophore linkage photosynthesis,” “light reaction oxidation‐reduction process,” “light harvesting in photosystem I” and “hydrogen peroxide metabolic process,” which related to photosynthesis and ROS response were enriched. All the significantly enriched GO terms were analyzed by REVIGO to further identify representative nonredundant GO terms (Figure 5). The expression of most of these significant enriched genes was induced under the shade conditions, for example, many photosynthesis‐related genes were upregulated in GO terms such as “photosystem I,” “photosynthetic membrane,” “chloroplast thylakoid photosystem II” and “photosynthesis, light harvesting” (Figure 6). These results suggested that genes involved in these GO terms might play important roles in the response to shade stress. During this process, *S*. *canadensis* might respond to shading stress by regulating the expression of these functional genes.

### 3.5 Pathway enrichment analysis

**Figure 7:**
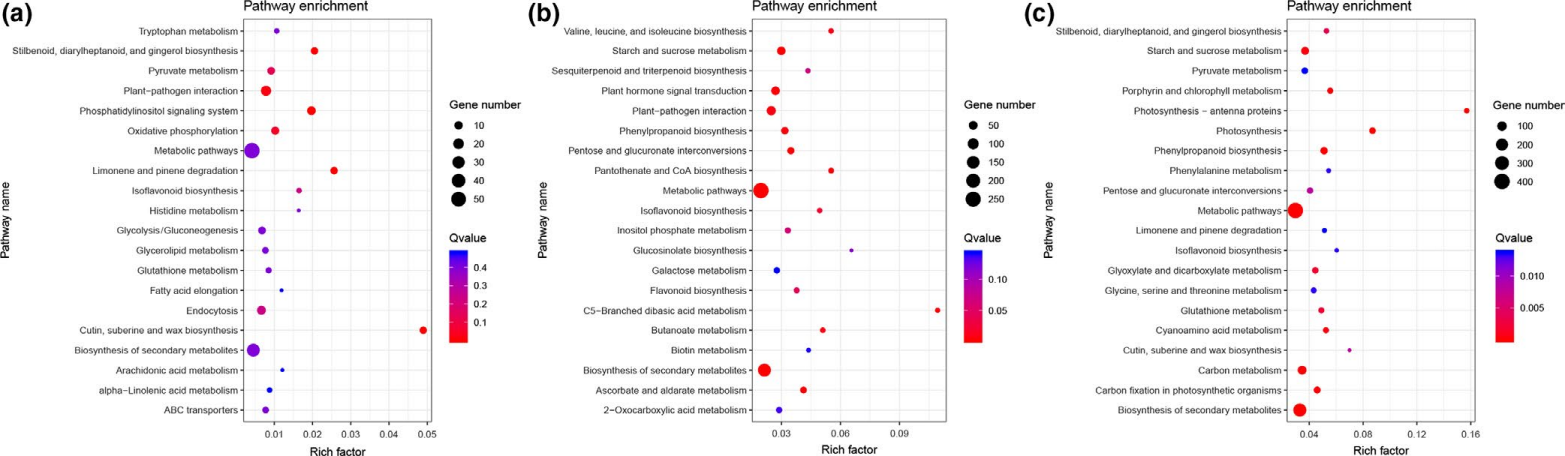
Top 20 enriched KEGG pathways among the annotated DEGs across three comparisons. (a) L_1_ versus L group; (b) L_2_ versus L group; and (c) L_3_ versus L group. The *y*‐axis on the left represents KEGG pathways, and the *x*‐axis indicates the enrichment factor. Low *Q*‐values are shown in red, and high *Q*‐values are shown in blue. The bubble size represents the number of enrichment genes in each pathway

**Figure 8:**
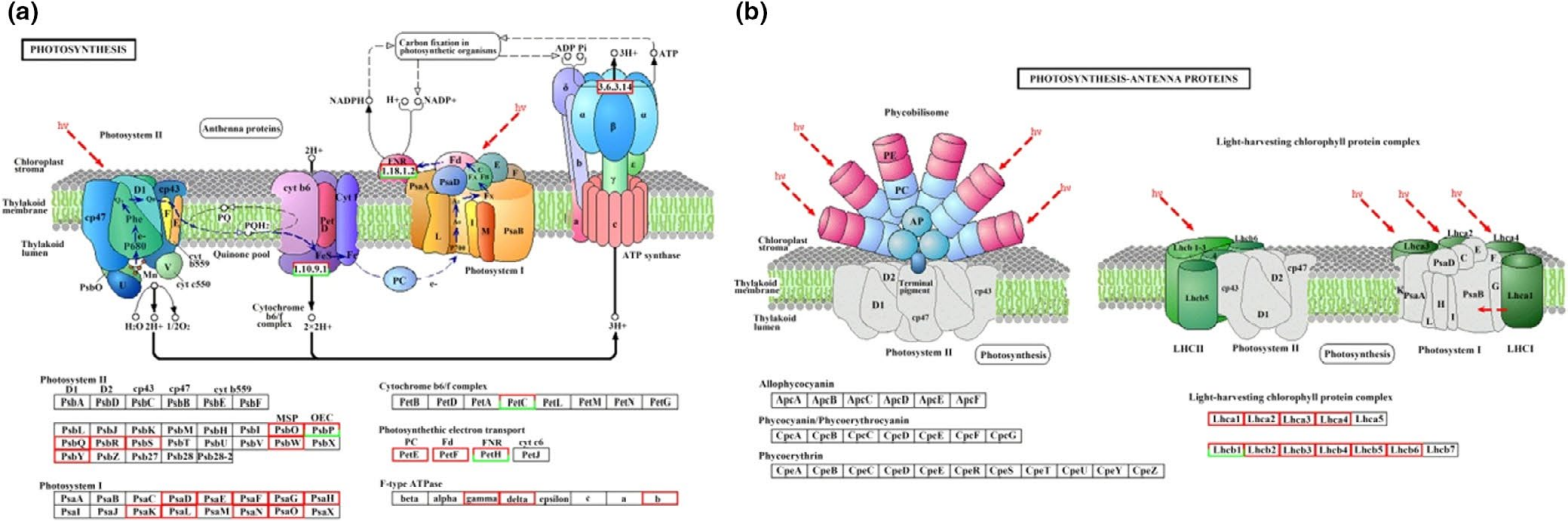
DEGs enriched in photosynthesis‐related pathways. (a) DEGs enriched in “photosynthesis” pathway; (b) DEGs enriched in “photosynthesis–antenna proteins” pathway. Red indicates upregulated genes, and green indicates downregulated genes. Semired and semigreen indicates genes including both upregulated and downregulated

According to the KEGG pathway analysis, 81, 117, and 125 pathways were enriched in L_1_ versus L, L_2_ versus L, and L_3_ versus L, respectively. The top 20 enriched pathways in L_1_ versus L (Figure 7a), L_2_ versus L (Figure 7b), and L_3_ versus L (Figure 7c) were ranked by Q‐value. Most of genes in these pathways such as “photosynthesis” (Figure 8a), “photosynthesis—antenna proteins” (Figure 8b), “phenylpropanoid biosynthesis,” “carbon metabolism,” “carbon fixation in photosynthetic organisms,” “starch and sucrose metabolism,” “stilbenoid, diarylheptanoid and gingerol biosynthesis,” “isoflavonoid biosynthesis,” “flavonoid biosynthesis” and “plant hormone signal transduction” were significantly enriched (Table S4). The KEGG enrichment analysis indicated that the number of significant enrichment pathways increased as the light intensity was reduced, and the DEGs that were significantly enriched in these pathways are likely to play essential roles in coping with shading stress in *S. canadensis*.

### 3.6 DEGs related to photosynthesis

**Figure 9:**
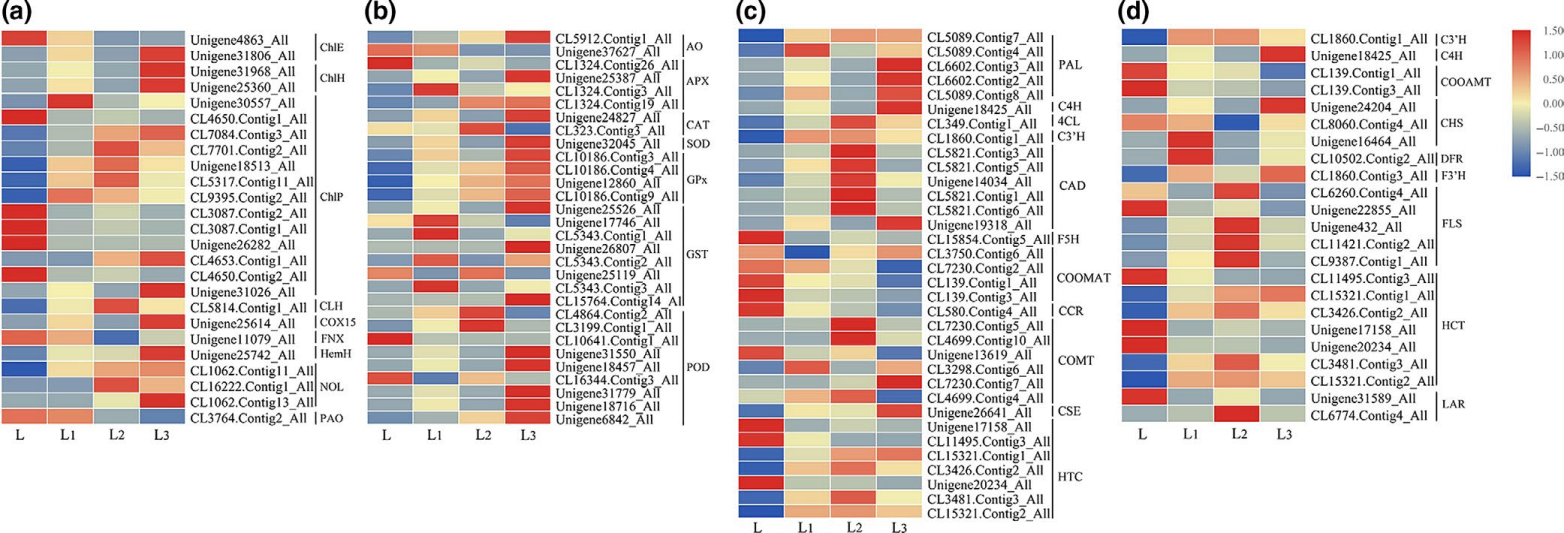
Expression profile clustering in various treatments. (a) Photosynthetic pigment; (b) flavonoid biosynthesis; (c) glutathione metabolism; and (d) peroxisome pathway

Many DEGs related to the photosynthesis process were collected. For example, photosystem I reaction center subunit‐related genes (*Psa*), such as the *PsaD*, *PsaE*, and *PsaH* gene, and photosystem II core complex protein‐related genes (*Psb*), such as the *PsbO*, *PsbQ,* and *PsbR* gene, were differentially expressed. Other photosynthesis‐related DEGs were as follows: cytochrome b6 complex and photosynthetic electron transport genes (*Pet*), such as *PetC*, *PetE,* and *PetF* gene; F‐type ATPase synthesis‐related genes, such as the ATP‐gamma gene (Figure 8a); light‐harvesting chlorophyll protein complex genes (*Lhc*), such as the *Lhca1*, *Lhca2*, and *Lhcb2* gene (Figure 8b); and carbon fixation process‐related genes, such as the ribulose‐bisphosphate carboxylase gene (*RBLC*), phosphoribulokinase gene (*PRK*), and glyceraldehyde‐3‐phosphate dehydrogenase gene (*NADP*). Furthermore, some Chl‐related genes, such as the *ChlP*, *ChlH,* and *HemH* gene, were also differentially expressed in the shade condition groups when compared with the control (Figure 9a). The number of these photosynthesis‐related genes increased with reduced light intensity. These results suggested that *S*. *canadensis* may cope with shading stress by regulating the expression of photosynthesis‐related genes.

### 3.7 Antioxidant enzyme‐related DEGs after shading

In this study, some antioxidant enzyme‐related genes, such as *SOD*, *CAT,* and *POD*, were differentially expressed in the shade conditions compared with the control, and most of them were upregulated (Figure 9b). In addition, other related DEGs which can encode antioxidant enzymes were also collected. For example, four glutathione peroxidase genes (*GPx*) encoding enzymes catalyzing the conversion of glutathione (GSH) to glutathione disulfide (GSSG) were upregulated. Among four ascorbate peroxidase genes (*APX*) encoding enzymes catalyzing the conversion of ascorbate to dehydroascorbate, three were upregulated and one downregulated. Of eight glutathione S‐transferase (*GST*) genes encoding enzymes catalyzing the conversion of glutathione (GSH) to R‐S‐glutathione, six were upregulated and two downregulated. Two ascorbate oxidase (*AO*) genes encoding enzymes catalyzing the conversion of dehydroascorbate to ascorbate, one was upregulated and another downregulated. These DEGs could encode related antioxidant enzymes in response to shading stress. Furthermore, the number of antioxidant enzyme‐related DEGs increased as the light intensity was reduced, and most were upregulated. These results indicated that the activity of antioxidant enzymes might be enhanced in *S*. *canadensis* in response to shade stress.

### 3.8 DEGs enriched in secondary metabolism‐related pathways

According to the KEGG pathway enrichment analysis, many DEGs from the three compared groups were enriched in secondary metabolism‐related pathways, such as “phenylpropanoid biosynthesis” and “flavonoid biosynthesis.” The number of DEGs in these two pathways increased with the reduction of light intensity and was significantly enriched in L_2_ versus L and L_3_ versus L. In contrast, the proportion of downregulated genes among these three comparisons increased in the “phenylpropanoid biosynthesis” pathway, in which 34 DEGs were collected and most were upregulated in the three shade treatment groups compared with the L group, such as the *PAL*, *C4H*, *4Cl,* and *C3′H* genes (Figure 9c). In the “flavonoid biosynthesis” pathway, 23 DEGs were collected, and most of these DEGs were also upregulated in the three shade treatment groups compared with the L group, such as the *CHS*, *DFR*, *F3′H,* and *FLS* genes (Figure 9d). In addition, some of these secondary metabolite synthesis‐related DEGs in this pathway in the L_3_ versus L group were downregulated compared with L_2_ versus L. These DEGs will respond to changes via induction or suppression in response to different levels of shading. These results indicated that shading stress might affect the secondary metabolism level in *S. canadensis*.

### 3.9 Gene expression network analysis of shade stress‐related DEGs

**Figure 10:**
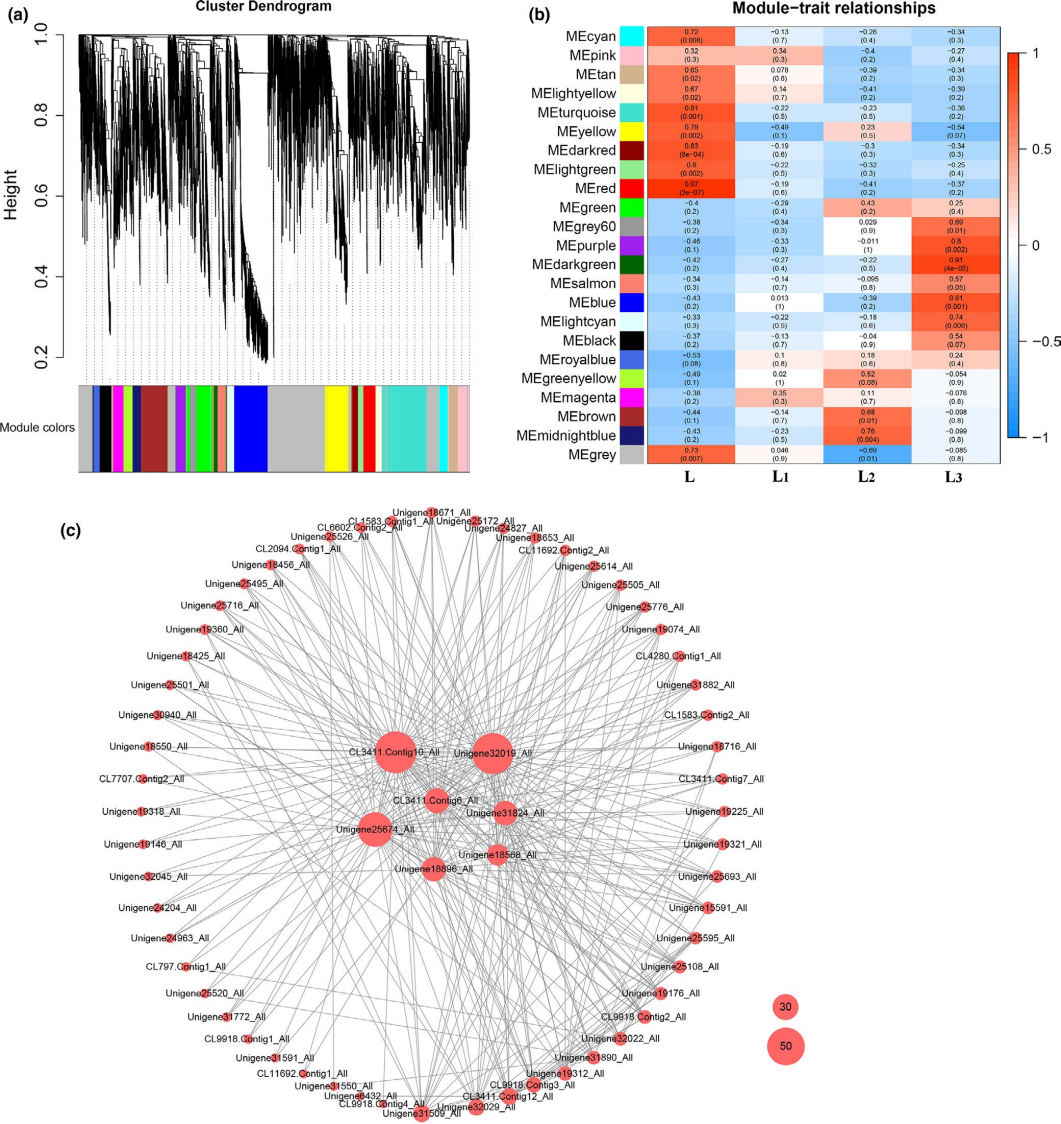
Gene expression network analysis. (a) Clustering dendrogram of genes, with dissimilarity based on topological overlap, together with assigned merged module colors and theory final module colors. (b) Module–trait associations. Each row corresponds to a module eigengene, column to a trait. Each cell contains the corresponding correlation and *p*‐value. The table is color‐coded by correlation according to the color legend. (c) Interaction analysis of the selected DEGs. The bubble size represents connect degree of each gene

In this study, 3,466 DEGs from three comparison groups were analyzed by WGCNA. These DEGs were parsed into 23 gene expression modules, with the number of genes in each module ranging from 43 to 871 (Figure 10a). The module–trait correlation analysis showed five significant coregulatory modules (correlation coefficient > 0.8, *p* < .01), with three in the L group shown in turquoise, dark red, and red and two in the L_3_ group shown in dark green and blue (Figure 10b). Therefore, the genes in the dark green and blue module may be vital for the L_3_ group. A total of 43 DEGs and all of them were upregulated in L_3_ versus L, which clustered in the dark green module. These DEGs include plant hormone signal transduction process‐related genes, such as *BSK* (BR‐signaling kinase) gene and *ARF* (auxin response factor) gene, and antioxidant‐related genes such as *AO* which encode ascorbate oxidase. A total of 297 DEGs, 267 upregulated and 30 downregulated in L_3_ versus L, were clustered in the blue module. Most of these DEGs were photosynthesis‐related genes, such as *Lhc*, chlorophyll biosynthesis, chloroplast thylakoid membrane, and carbon fixation. Meanwhile, antioxidant enzymes‐related genes, such as *POD*, *SOD*, *CAT*, *APX,* and *GST,* and secondary metabolism‐related genes, such as *PAL*, *C4H*, *CAD* and *CHS*, were also clustered into this module. In addition, some transcription factor genes, such as *EREBP*, *ERF*, *WRKY*, *TIFY,* and *bHLH*, may encode corresponding TFs and play a regulatory role in this module. Based on the network analysis for the blue module, the top 300 paired DEGs with a weight value above 0.71 were chosen for exhibition (Figure 10c). Some hub genes with a connect degree ≥7 significantly expressed genes were related to photosynthesis and present in the network, such as *Lhcb1*, *Lhcb2*, *Lhca1*, *Lhcb5*, and *RBLC* gene (Table 2). These hub genes may play a key role in the response of *S*. *canadensis* to shade stress.

**Table 2:** The annotation of the hub genes identified by WGCNA. The node represents collected gene, and the number represents the number of connect degree

Node	Number	Gene annotation	GO term
CL3411.Contig10_All	57	Light‐harvesting complex II chlorophyll *a*/*b* binding protein 1	Photosynthesis, light harvesting in photosystem II
Unigene32019_All	56	Light‐harvesting complex II chlorophyll *a*/*b* binding protein 2	Photosynthesis, light harvesting in photosystem II
Unigene25674_All	45	Light‐harvesting complex II chlorophyll *a*/*b* binding protein 5	Photosynthesis, light harvesting in photosystem II
CL3411.Contig6_All	29	Light‐harvesting complex II chlorophyll *a*/*b* binding protein 1	Photosynthesis, light harvesting in photosystem II
Unigene18896_All	28	Light‐harvesting complex II chlorophyll *a*/*b* binding protein 1	Photosynthesis, light harvesting in photosystem II
Unigene31824_All	28	Light‐harvesting complex I chlorophyll *a*/*b* binding protein 1	Photosynthesis, light harvesting in photosystem I
Unigene18568_All	23	Ribulose‐bisphosphate carboxylase/oxygenase activase	ATP binding
Unigene31509_All	16	Glyceraldehyde‐3‐phosphate dehydrogenase	Oxidation‐reduction process
Unigene32029_All	15	Light‐harvesting complex I chlorophyll *a*/*b* binding protein 3	Photosynthesis, light harvesting in photosystem I
CL3411.Contig12_All	15	Light‐harvesting complex II chlorophyll *a*/*b* binding protein 1	Photosynthesis, light harvesting in photosystem II
CL9918.Contig3_All	13	Ribulose‐bisphosphate carboxylase	Ribulose‐bisphosphate carboxylase activity
Unigene19312_All	11	Photosystem II oxygen‐evolving enhancer protein 3	Photosystem II
Unigene31890_All	10	Photosystem I subunit PsaN	Photosystem I
CL9918.Contig2_All	10	Ribulose‐bisphosphate carboxylase	Ribulose‐bisphosphate carboxylase activity
Unigene32022_All	10	Photosystem I subunit V	Photosystem I
Unigene25108_All	9	Fructose‐bisphosphate aldolase	Fructose‐bisphosphate aldolase activity
Unigene19176_All	9	Light‐harvesting complex II chlorophyll *a*/*b* binding protein 6	Photosynthesis, light harvesting in photosystem II
Unigene25595_All	8	Photosystem I subunit XI	Photosystem I
Unigene25693_All	8	Light‐harvesting complex II chlorophyll *a*/*b* binding protein 4	Photosynthesis, light harvesting in photosystem I
Unigene15591_All	8	Fructose‐bisphosphate aldolase	Fructose‐bisphosphate aldolase activity
Unigene19321_All	7	Photosystem I subunit PsaO	Photosystem I

### 3.10 Gene expression validation by qRT‐PCR

**Figure 11:**
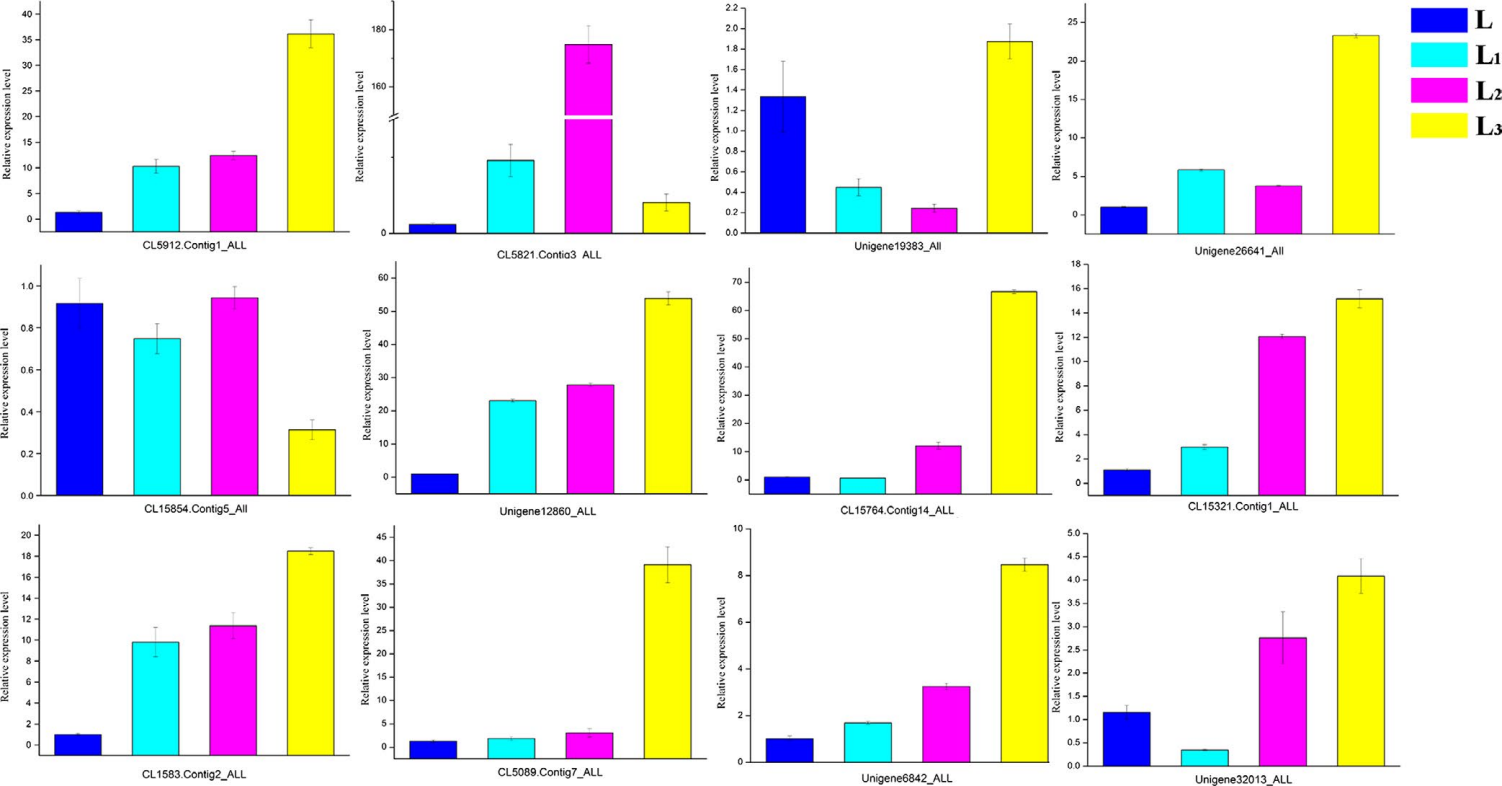
The result of qRT‐PCR for randomly selected genes. The blue, cyan, rose, and yellow bar represent the expression of selected genes in L, L_1_, L_2,_ and L_3_ group respectively

The gene expression results obtained from the high‐throughput sequencing were validated by quantitative reverse transcription‐PCR (qRT‐PCR) analysis. A total of 12 randomly selected key candidate genes from each of four different treatment groups were used for validation. As anticipated, the qRT‐PCR results for the different groups (Figure 11) were consistent with the sequencing data. The primer sequences used in this study are listed in Table S5.

## 4 DISCUSSION

Previous studies have elaborated that invasive species consistently show adaptive potential and rapidly adapt to novel environments (Bertelsmeier & Keller, 2018; Liu, Li, & Xiong, 2015). Furthermore, these species often have the ability to express different phenotypes in response to changing or novel environmental conditions and display higher plasticity, even in low‐resource systems (Funk, 2008). *S*. *canadensis*, an invasive plant, could adapt to low‐light conditions in a shade stress environment by adjusting the morphology, growth, photosynthetic physiology, and other characteristics, and it exhibits a certain plasticity in response to low light intensity (Du et al., 2017). However, the regulatory mechanisms of shade stress acclimation in *S*. *canadensis* have not been determined. In the present study, the gene expression and morphological traits of *S*. *canadensis*, cultivated with different levels of shade conditions, were used to understand the regulatory mechanisms that contribute to shade stress.

### 4.1 Alteration of phenotype may facilitate the growth of *S. canadensis* under shade condition

Leaf area and leaf angle insertion are considered to be responses to the shading environment and increase with shade application (Ajmi et al., 2018). Under limiting irradiance, leaves developed large areas to capture more light sources and help the plants maintain better performance (Liu et al., 2016; Yang, Sun, Zhang, Cochard, & Cao, 2014). Furthermore, leaf insertion angles are important in determining the transmission of radiation for vegetation canopies, and the direction of leaves depends greatly on the light exposure (Raabe, Pisek, Sonnentag, & Annuk, 2015). Under shade stress, to absorb more light, the plant leaf angle insertion can be extended to 100°, while under normalized light, to avoid excess sunlight, the leaf angle insertion always ranges from 30° to 60° (Larbi et al., 2015). In our study, all the plants under shading treatment had a larger leaf area and leaf angle insertion than those cultivated under natural light. Such a strategy will assist the capture of light energy by *S*. *canadensis* and improved acclimation to the shading environment.

### 4.2 Alteration of physiology may contribute to the shade tolerance of *S. canadensis*

Chlorophyll is the most important photosynthetic pigment in photosynthetic processes, and the chlorophyll content is always significantly affected by shade stress (Alridiwirsah, Harahap, Akoeb, & Hanum, 2018). Under shade stress, plants increase their light use efficiency by increasing leaf chlorophyll *a* and chlorophyll *b*, total chlorophyll content, and decreasing chlorophyll *a*/*b* ratios (Dai et al., 2009; Gregoriou, Pontikis, & Vemmos, 2007), and if the available light source is reduced in a shade environment for photosynthesis, the chlorophyll content will be increased (Muhidin et al., 2018). Our results revealed a significantly increased chlorophyll content (chl *a*, chl *b*, and chl *a* + *b*) in the different treatment groups. These results were consistent with the linear increase in shade stress, concurrent with a significant decrease in the chl *a*/*b* ratio. The carotenoid content increased in the three treatment groups with no significant differences among them. The ratio of carotenoid content with total chlorophyll was decreased with the reduction of light radiation. This phenomenon indicates that the photosynthesis system in *S*. *canadensis* has a wide range of strategies to adapt to shading stress and increase its light use efficiency by regulating the content of chlorophyll.

### 4.3 Photosynthesis‐related genes in *S. canadensis* play a vital role in the shade environment

To maximize light use for photosynthesis, plants possess fully developed strategies based on short‐ and long‐term responses to adapt to low light intensities (Mathur, Jain, & Jajoo, 2018). Photosynthesis is the key function for plant growth and is highly sensitive to light conditions. In photosynthetic systems, the pigment protein complexes photosystem I and photosystem II are interconnected through the cytochrome (Cyt) *b_6_f* complex and electron transfer chain (Mamedov, Govindjee, Nadtochenko, & Semenov, 2015). Photosystem II is a protein complex embedded in thylakoid membranes of higher plants and able to absorb light and split water. The core complex subunits, such as PsbB, PsbC, PsbR, PsbO, PsbP, and PsbQ proteins, are also called oxygen‐evolving enhancer (OEE) proteins, with main functions of enhancing the efficiency of the oxygen‐evolving complex (Bricker, Roose, Fagerlund, Frankel, & Eaton‐Rye, 2012; Silveira & Carvalho, 2016). Similar to photosystem II, the photosystem I core complex contains reaction center subunits (PsaA and PsaB), binding subunits (PsaG, PsaK, PsaH, PsaL, PsaO, and PsaP), ferredoxin docking subunits (PsaD and PsaE), plastocyanin docking subunits (PsaF and PsaN), and FeS apoprotein (PsaC; Kouril et al., 2014). Furthermore, ATP synthase subunits (α, β, γ, and δ), cytochrome *b_6_f* (PetB, PetD, PetA, and PetC), ferredoxin (Fdx) complex, and ribulose‐bisphosphate carboxylase large chain (RbcL) involved in the Calvin cycle can play important roles in the photosynthesis process (Silveira & Carvalho, 2016). The results for the three comparison groups revealed significant differences in photosynthesis‐related GO terms and KEGG pathways. In the present study, most of the photosynthesis‐related DEGs were enriched in the “photosynthesis,” “photosynthesis‐antenna proteins,” and “carbon fixation in photosynthetic organisms” pathways. Moreover, the “photosynthesis” pathway contains photosystem II, photosystem I, cytochrome b6/f complex, electron transport, and ATP synthase‐related genes. Our result showed that most DEGs were upregulated and enriched in photosynthesis II, such as *PsbO*, *PsbP*, *PsbQ*, *PsbR,* and *PsbW*, and photosynthesis I such as *PsaD*, *PsaE*, *PsaF*, *PsaH*, and *PsaK*. These upregulated DEGs indicated that *S*. *canadensis* could enhance the expression of photosynthesis II‐ and photosynthesis I‐related genes to adapt to low‐light conditions. Cytochrome *b_6_f*, Fdx complex, ATP synthase, and RbcL‐related DEGs, such as *PetC*, *PetF*, *PetH*, ATP synthase γ, δ, and b, were also related upregulated DEGs that were enriched in the “photosynthesis” pathway. These results indicated that enriched DEGs might be vital for *S*. *canadensis* to resist shade stress conditions.

The pathway of “photosynthesis‐antenna proteins” includes light‐harvesting chlorophyll protein complex I (LHCI) and II (LCHII)‐related genes encoding subunits consisting of the photosystem I‐antenna and photosystem II‐antenna complex, respectively. Photosystem II‐antenna complexes include major antenna complexes (LHCII), such as Lhcb1, Lhcb2, and Lhcb3 proteins, which bind several pigment molecules, such as chlorophyll *a*/*b*, zeaxanthin (Zx), and lutein (Lut), playing a vital role in photosystem II (van Amerongen & Croce, 2013). The photosystem I‐antenna (LHCI), such as Lhca1, Lhca2, and Lhca3, contains LHC trimers that are also essential for coping with different light conditions (Puthiyaveetil, Ibrahim, & Allen, 2012). In the present study, the expression of LHCI‐related DEGs, such as *Lhca1*, *Lhca2*, *Lhca3*, and *Lhca4*, and LHCII‐related DEGs, such as *Lhcb1*, *Lhcb2*, *Lhcb3*, *Lhcb4* and *Lhcb5*, was upregulated under shade conditions. This result suggests that *S*. *canadensis* can adapt to low‐light conditions by enhancing the expression of LHC‐related genes under shading stress.

Most of enzymes, such as phosphoribulokinase (PRK), phosphoglyceratekinase (PGK), RuBP carboxylase (rubisco), fructose‐bisphosphatase aldolase (ALDO), and glyceraldehyde phosphate dehydrogenase (GAPDH), plays an important role in the carbon fixation process (Huang et al., 2017; Silveira & Carvalho, 2016). Many DEGs, such as *rbcL*, *PGK*, *GAPDH*, and *ALDO*, were upregulated, which might enhance carbon synthesis in *S*. *canadensis* under shade conditions.

### 4.4 Expression of ROS response enzyme‐related genes maintains growth health under shade stress

Reactive oxygen species always act as important molecules and play pivotal roles in plant for abiotic stress (Chan, Yokawa, Kim, & Song, 2016; Li et al., 2018; Ma et al., 2011; Qi et al., 2018). Furthermore, ROS were also characterized as important signaling molecules that mediate plant hormone signaling transduction process, such as mitogen‐activated protein kinase (Wang, Du, Li, Ren, & Song, 2010; Wang, Du, Zhao, Miao, & Song, 2013) and hormone signaling (Chen, Li, Li, Song, & Miao, 2017; Dong, Wei, & Chen, 2013; Song, Miao, & Song, 2014). The activities of antioxidant enzymes, such as superoxide dismutase (SOD), catalase (CAT), and peroxidase (POD), were significantly changed under different light conditions in *Camellia sinensis* (Wu et al., 2016). When plants are exposed to high irradiance conditions, the levels of antioxidant enzymes such as POD, SOD, and CAT are significantly altered (Shao et al., 2014). In this study, with increasing shade stress, a number of antioxidant enzyme‐related DEGs also increased, such as *POD*, *SOD*, *CAT*, *APX*, and *GPx*. Therefore, under shade stress, antioxidant enzyme activity may be altered in *S*. *canadensis* by regulating the expression of related genes to scavenge ROS, thereby maintaining normal plant growth.

### 4.5 TF gene regulation may promote acclimation to shade stress in *S. canadensis*

To adapt to adverse environmental conditions, many plants have developed different mechanisms in response to abiotic stresses. Most transcription factors (TFs) can act as regulatory proteins that play an important role in these mechanisms (Wang, Yang, et al., 2018). For example, the TIFY family is a novel plant‐specific gene family that participates in the regulation of various plant‐specific biologic processes (Sun et al., 2017). The AP2/EREBP superfamily is one of the largest and most conserved gene families in plants, and expansion of the AP2/EREBP superfamily may have enhanced the wide adaptability of cotton (Liu & Zhang, 2017). Furthermore, the WRKY and MYB TF families play a vital role in diverse regulatory and multiple stress response processes in plants (Li et al., 2019; Wang, Ren, et al., 2019; Wang, Yue, et al., 2019). In this study, *AP2/EREBP* and *TIFY* genes predominated in the three comparison groups of differentially expressed TF‐coding genes followed by others, such as *MYB‐*, *NAC‐*, *WRKY‐,* and *bHLH* TF‐coding genes, which were also differentially expressed and mostly upregulated in each group. Therefore, the *AP2/EREBP* and *TIFY* TF genes may play an important role in the process of shading stress in *S*. *canadensis*, and they may promote an increase in tolerance to stressful environments.

## 5 CONCLUSIONS

Invasive species consistently display the trait of phenotypic plasticity to adapt to various environments. The results from this study confirm that shade levels strongly affect phenotypic changes and physiological responses in *S*. *canadensis*. The expression levels of photosynthesis and antioxidant enzyme‐related genes were altered in response to shade stress. This phenomenon suggests that *S*. *canadensis* has enhanced photosynthesis and ROS scavenging abilities to absorb large amounts of light and ensure normal growth. While the expression of secondary metabolism‐related genes was also affected under shade stress, this phenomenon was indicative of an adaptive strategy to retain fitness under harsh conditions. The expression network analyses revealed the important roles of candidate genes for shading tolerance in *S. canadensis*. This shading tolerance might facilitate *S*. *canadensis* to adapt to various environments and successfully develop into an invasive species.

## CONFLICT OF INTEREST

The authors declare no conflict of interest.

## AUTHOR CONTRIBUTIONS

**Miao Wu: **Conceptualization (lead); data curation (lead); formal analysis (lead); investigation (lead); methodology (lead); project administration (lead); resources (lead); software (lead); supervision (lead); validation (lead); visualization (lead); writing – original draft (lead); writing – review and editing (lead). **Zeyu Li: **Data curation (supporting); methodology (supporting); resources (supporting); software (supporting). **Jianbo Wang: **Conceptualization (lead); data curation (lead); formal analysis (lead); funding acquisition (lead); investigation (lead); methodology (lead); project administration (lead); resources (lead); software (equal); supervision (lead); validation (equal); visualization (lead), writing – original draft (equal); writing – review and editing (equal).

## Supporting information

Fig S1

Fig S2

Table S1

Table S2

Table S3

Table S4

Table S5

Supplementary Material

## Data Availability

The mRNA‐seq data as fastq files from the experiment of *S. canadensis* were deposited in NCBI SRA database under the accession number PRJNA577291.
