# Supplementary figures and images for: Transcriptional analyses reveal the molecular mechanism governing shade tolerance in the invasive plant Solidago canadensis
Source: Ecol Evol. 2020 Mar 24;10(10):4391–406. doi: 10.1002/ece3.6206 (PMC7246212; doi:10.1002/ece3.6206)

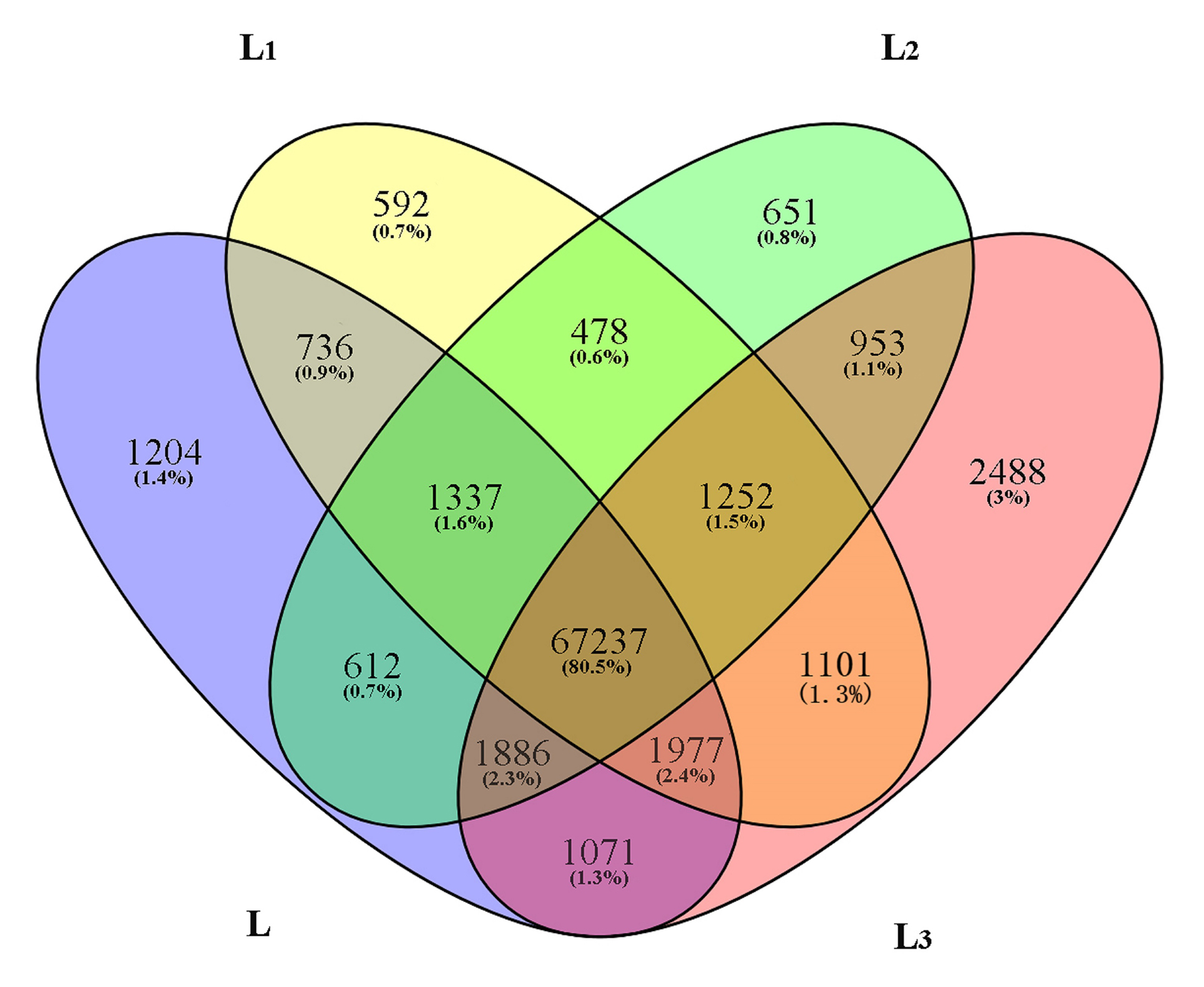

Supplement: Supplementary file 1 — Fig S1 [file ECE3-10-4391-s001.jpg]

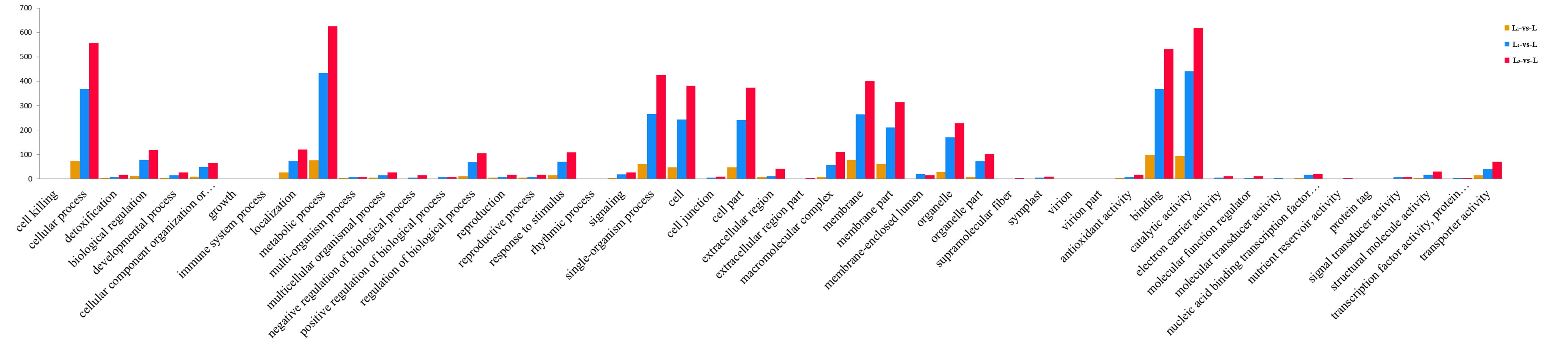

Supplement: Supplementary file 2 — Fig S2 [file ECE3-10-4391-s002.jpg]
