# Supplementary material for: Transcriptional analyses reveal the molecular mechanism governing shade tolerance in the invasive plant Solidago canadensis
Source: Ecol Evol. 2020 Mar 24;10(10):4391–406. doi: 10.1002/ece3.6206 (PMC7246212; doi:10.1002/ece3.6206)
Supplement: Supplementary file 3 — Table S1 [file ECE3-10-4391-s003.docx]

Table S1．Pigment content in *S. canadensis* under different shade conditions.

| Groups | Chl a/mg·g^-1^ | Chl b/mg·g^-1^ | Carotenoid/mg·g^-1^ | Total Chl/mg·g^-1^ | Chl a/Chl b | Carotenoid/Total Chl |
| --- | --- | --- | --- | --- | --- | --- |
| L | 0.92 | 0.28 | 0.22 | 1.20 | 3.29 | 0.18 |
| L_1_ | 1.33 | 0.44 | 0.29 | 1.77 | 3.02 | 0.16 |
| L_2_ | 1.53 | 0.58 | 0.30 | 2.11 | 2.64 | 0.14 |
| L_3_ | 1.67 | 0.71 | 0.28 | 2.38 | 2.35 | 0.12 |
