# Supplementary material for: Transcriptional analyses reveal the molecular mechanism governing shade tolerance in the invasive plant Solidago canadensis
Source: Ecol Evol. 2020 Mar 24;10(10):4391–406. doi: 10.1002/ece3.6206 (PMC7246212; doi:10.1002/ece3.6206)
Supplement: Supplementary file 5 — Table S3 [file ECE3-10-4391-s005.docx]

| Table S3: The significantly enriched GO terms of three compared groups. | | | |
| --- | --- | --- | --- |
| Comparion groups | Cellular component | Molecular function | Biological processes |
| L_1_-vs-L | membrane | primary active transmembrane transporter activity | lipid transport |
|  | extracellular region | P-P-bond-hydrolysis-driven transmembrane transporter activity | cuticle hydrocarbon biosynthetic process |
|  | cell wall |  | cuticle development |
|  | external encapsulating structure |  | cellular alkane metabolic process |
|  |  |  | alkane biosynthetic process |
|  |  |  | lipid localization |
|  |  |  | cellular process involved in reproduction in multicellular organism |
|  |  |  | pollen sperm cell differentiation |
| L_2_-vs-L | intraciliary transport particle | sucrose alpha-glucosidase activity |  |
|  | intraciliary transport particle A | beta-fructofuranosidase activity |  |
|  | amyloplast | alpha-glucosidase activity |  |
|  |  | sulfite reductase activity |  |
|  |  | oxidoreductase activity, acting on a sulfur group of donors, iron-sulfur protein as acceptor |  |
|  |  | sulfite reductase (ferredoxin) activity |  |
|  |  | carboxypeptidase activity |  |
| L_3_-vs-L | photosystem | tetrapyrrole binding | photosynthesis |
|  | photosystem I | chlorophyll binding | photosynthesis, light harvesting |
|  | photosynthetic membrane | oxidoreductase activity | protein-chromophore linkage |
|  | extracellular region | metal ion binding | photosynthesis, light reaction |
|  | thylakoid | cation binding | oxidation-reduction process |
|  | thylakoid part | pigment binding | photosynthesis, light harvesting in photosystem I |
|  | cell wall | heme binding | generation of precursor metabolites and energy |
|  | external encapsulating structure | aspartyl esterase activity | hydrogen peroxide catabolic process |
|  | thylakoid membrane | germacrene-A synthase activity | cell wall organization |
|  | chloroplast thylakoid membrane | peroxidase activity | pectin catabolic process |
|  | plastid thylakoid membrane | pectinesterase activity | hydrogen peroxide metabolic process |
|  | chloroplast thylakoid | sesquiterpene synthase activity | external encapsulating structure organization |
|  | plastid thylakoid |  | cell wall organization or biogenesis |
|  | membrane protein complex |  |  |
|  | photosystem II |  |  |
|  | apoplast |  |  |
|  | plastoglobule |  |  |
|  | photosystem I reaction center |  |  |
