# Supplementary material for: Transcriptional analyses reveal the molecular mechanism governing shade tolerance in the invasive plant Solidago canadensis
Source: Ecol Evol. 2020 Mar 24;10(10):4391–406. doi: 10.1002/ece3.6206 (PMC7246212; doi:10.1002/ece3.6206)
Supplement: Supplementary file 6 — Table S4 [file ECE3-10-4391-s006.docx]

| Table S4: The significantly enriched KEGG pathway of three compared groups. | | | |
| --- | --- | --- | --- |
| Comparion group | Pathway | Gene number | Qvalue |
| L_1_-vs-L | Cutin, suberine and wax biosynthesis | 7 | 6.50E-05 |
|  | Phosphatidylinositol signaling system | 11 | 2.07E-04 |
|  | Limonene and pinene degradation | 7 | 1.47E-03 |
|  | Stilbenoid, diarylheptanoid and gingerol biosynthesis | 7 | 4.35E-03 |
|  | Plant-pathogen interaction | 18 | 2.03E-02 |
| L_2_-vs-L | Starch and sucrose metabolism | 47 | 0.00239 |
|  | Biosynthesis of secondary metabolites | 165 | 0.00239 |
|  | C5-Branched dibasic acid metabolism | 7 | 0.00239 |
|  | Metabolic pathways | 264 | 0.00239 |
|  | Ascorbate and aldarate metabolism | 19 | 0.00429 |
|  | Pentose and glucuronate interconversions | 24 | 0.00554 |
|  | Plant hormone signal transduction | 47 | 0.00554 |
|  | Pantothenate and CoA biosynthesis | 11 | 0.00554 |
|  | Phenylpropanoid biosynthesis | 28 | 0.00675 |
|  | Plant-pathogen interaction | 57 | 0.00826 |
|  | Butanoate metabolism | 10 | 0.01235 |
|  | Valine, leucine and isoleucine biosynthesis | 9 | 0.01235 |
|  | Isoflavonoid biosynthesis | 9 | 0.02418 |
|  | Flavonoid biosynthesis | 12 | 0.04481 |
| L_3_-vs-L | Metabolic pathways | 400 | 4.99E-12 |
|  | Biosynthesis of secondary metabolites | 255 | 1.39E-11 |
|  | Photosynthesis | 30 | 4.22E-09 |
|  | Photosynthesis - antenna proteins | 17 | 4.35E-09 |
|  | Phenylpropanoid biosynthesis | 45 | 2.17E-06 |
|  | Carbon metabolism | 82 | 2.12E-04 |
|  | Carbon fixation in photosynthetic organisms | 36 | 2.96E-04 |
|  | Porphyrin and chlorophyll metabolism | 24 | 3.47E-04 |
|  | Starch and sucrose metabolism | 58 | 5.45E-04 |
|  | Cyanoamino acid metabolism | 24 | 6.99E-04 |
|  | Glyoxylate and dicarboxylate metabolism | 29 | 2.00E-03 |
|  | Glutathione metabolism | 23 | 2.17E-03 |
|  | Stilbenoid, diarylheptanoid and gingerol biosynthesis | 18 | 3.98E-03 |
|  | Pentose and glucuronate interconversions | 28 | 8.21E-03 |
|  | Cutin, suberine and wax biosynthesis | 10 | 8.21E-03 |
|  | Glycine, serine and threonine metabolism | 21 | 1.33E-02 |
|  | Isoflavonoid biosynthesis | 11 | 1.33E-02 |
|  | Phenylalanine metabolism | 13 | 1.33E-02 |
|  | Pyruvate metabolism | 32 | 1.37E-02 |
|  | Limonene and pinene degradation | 14 | 1.37E-02 |
|  | Nitrogen metabolism | 13 | 1.37E-02 |
|  | Glycolysis / Gluconeogenesis | 40 | 1.51E-02 |
|  | Flavonoid biosynthesis | 15 | 1.93E-02 |
|  | Oxidative phosphorylation | 31 | 2.31E-02 |
|  | alpha-Linolenic acid metabolism | 15 | 3.41E-02 |
|  | Glucosinolate biosynthesis | 5 | 4.53E-02 |
|  | Ascorbate and aldarate metabolism | 18 | 4.78E-02 |
