# Supplementary material for: Transcriptional analyses reveal the molecular mechanism governing shade tolerance in the invasive plant Solidago canadensis
Source: Ecol Evol. 2020 Mar 24;10(10):4391–406. doi: 10.1002/ece3.6206 (PMC7246212; doi:10.1002/ece3.6206)
Supplement: Supplementary file 8 — Supplementary Material [file ECE3-10-4391-s008.docx]

SUPPLEMENTARY DATA

Fig. S1 Venn diagram of the gene distribution among four groups. A total of 83,575 unigenes were identified, with 67,237 unigenes was detected in all four groups and 1204 unigenes that were unique to the L group, 592 to the L_1_ group, 651 to the L_2_ group, and 2488 to the L_3_ group.

Fig. S2 GO functional enrichment analysis of DEGs from the three comparison groups. The orange, blue and red color bar represent L_1_ vs. L, L_2_ vs. L and L_3_ vs. L group respectively. Most of DEGs were enriched into 49 GO terms, and the number of DEGs in each group increased with the increase of shading.

Table S1. Pigment content in *S. canadensis* under different shade conditions.

Table S2. The result of clean data in each library.

Table S3. The significantly enriched GO terms of three compared groups.

Table S4. KEGG pathway analysis of DEGs from the three comparison groups.

Table S5. The list of primer sequence.
